# Fock-Space Coupled Cluster Theory: Systematic Study of Partial Fourth Order Triples Schemes for Ionization Potential and Comparison with Bondonic Formalism

**DOI:** 10.3390/ijms21176199

**Published:** 2020-08-27

**Authors:** Suhita Basumallick, Sourav Pal, Mihai V. Putz

**Affiliations:** 1 Department of Chemistry, Indian Institute of Technology Bombay, Powai, Mumbai 400076, India; basumallick.suhita16@gmail.com; 2 Department of Chemical Sciences, Indian Institute of Science Education and Research Kolkata, Mohanpur, Nadia 741246, West Bengal, India; 3 Laboratory of Structural and Computational Physical-Chemistry for Nanosciences and QSAR, Biology-Chemistry Department, Faculty of Chemistry, Biology, Geography, West University of Timisoara, Pestalozzi Street No. 16A, 300115 Timisoara, Romania; 4 Laboratory of Renewable Energies-Photovoltaics, R&D National Institute for Electrochemistry and Condensed Matter, Street Dr. Aurel Podeanu No. 144, 300569 Timisoara, Romania

**Keywords:** ionization potential (IP), Fock-space coupled-cluster theory, perturbative triples, multi-reference coupled-cluster, bondonic formalism

## Abstract

In this paper, we have made a systematic study of partial fourth order perturbative schemes due to triples to compute the ionization potential within Fock-space multi-reference coupled-cluster theory. In particular, we have obtained computationally less expensive correlation schemes due to fourth order triples. Prototype examples have been considered to explore the efficacy of the approximate methods mentioned, while the bondonic formalism supporting the bonding phenomenology is also respectively for the first time here advanced.

## 1. Introduction

Photo-ionization of electrons is a very important step to transfer the energy of radiation to matter and thus plays a crucial role in physics and chemistry [1]. Ionization of main peaks is associated with ejection of electrons from the molecular orbitals and simple Koopman’s approximation is often inadequate to describe the process of ionization. While the satellite ionization peaks cannot be explained without electron correlation, as pointed out by Cederbaum and co-workers [2], the role of electron correlation and thus the correlated theories in the calculations of main peaks has been noted in several works [3]. For the main peaks, such correlated theories improve the main peaks significantly and very often without such correlation, even the qualitative ordering of ionization peaks does not come out correctly. Several correlated theories, configuration interaction, perturbation-based, Green’s function, equation-of-motion based theories have been used for *vertical ionization potential* (VIP) calculation. The ones, which can produce direct calculation of VIPs by cancellation of the common ground state energies, have been preferred [4,5]. Several quantum mechanical theories are able to describe these energies as well as microscopic interactions of the systems. At the same time, theories based on bondons provide the link to the extended systems [6,7]. The bondons describe chemical particles, which are associated with electrons implicated in the chemical bond as single, delocalized, or lone pair; they have orientated direction of movement and depend on a chemical field. The bondons can be found on the wave functions, on the covalent bonds, in dispersive–weak interactions, on the mechanism of action between ligand and receptor, and even on the ionic interactions.

Among several quantum mechanical theories, including electron correlation from first-principle, *coupled-cluster* (CC) theory [8] has been established as a state-of-the-art [8,9,10,11,12,13,14,15,16,17] theory for inclusion of what is known as “dynamic electron correlation”. It sums up selective perturbation terms up to infinite order through an exponential wave-operator [8]. Traditionally, CC theory is developed with exponential wave-operator acting on a single determinant reference. It works well when a particular determinant, like restricted Hartree–Fock (RHF), is dominant in the description of the wave function. However, there are cases where multiple determinants have large amplitudes in the configuration expansion of the wave-function. Example cases are excited states, molecules at stretched geometries, ionized or electron-attached states etc. These are the cases where static correlation also becomes dominant. For ionized states, such static correlation occurs due to the degeneracy of the occupied orbitals, where the reference consists of multiple determinants involving what are termed “active” holes. *Multi-reference* CC (MRCC) [18,19,20,21,22,23,24,25,26,27,28,29,30,31,32,33,34,35,36,37,38,39,40,41,42] has turned out to be a physically appealing theory to take care of both static and dynamic correlation. Dynamical correlation is described by the CC wave-operator satisfying Bloch equation or an equivalent equation, while the static correlation is described by a diagonalization of an effective Hamiltonian over the small reference space. Such effective Hamiltonian theories, first developed by Malrieu and co-workers [18], have been the cornerstone of effective Hamiltonian MRCC theories. These are further sub-divided into Hilbert- [19,20,21] and *Fock-space* (FS) [19,22,23,24,25,26,27,28,29,30,31,32,33,34,35,36,37,38,39,40,41,42] theories. Hilbert space theories have an associated cluster operator for every determinant in the reference, with each of these determinants acting as a vacuum. Thus, the cluster operators are hole-particle creation operators with respect to the corresponding vacuum. These are ideally used for potential energy surfaces and the state-selective versions of the Hilbert-space theories with some modifications are even more appropriate for energy surface of selected states. On the other hand, the Fock-space CC (FS-CC) is based on a common vacuum, which defines holes and particles. Exponential wave-operator acts on the reference function. Due to the common vacuum, the reference space contains determinants classified to have holes and particles which may be called “active”. The choice of active holes and particles often determines the success and convergence of FS-CC theories. The cluster operators have a more complicated structure than the Hilbert-space theory and contains destruction of the active holes and particles, along with other creation operators.

The FS-CC theories are ideally suited to the vertical *ionization potential* (IP), *electron affinity* (EA), and *excited state energies* (EE) [24,25,26,29,30,31,32,33] and are formulated such that the common correlation of the ground neutral molecule is cancelled. Thus, direct determination of these vertical energies is possible. By sacrificing the direct determination, the adiabatic calculations of the above quantities can also be done. In particular, the one active-hole reference, commonly denoted as (0.1) FS-sector, is very helpful to describe the main ionization peaks.

The FS-CC, in *singles and doubles approximations* (FS-CCSD), has been extensively studied [24,25,26,37,41,42]. FS-CC was pioneered by several studies from the groups of Mukherjee [19,22,23,28], Kaldor [34,35], Lindgren [40], Kutzelnigg [27], Bartlett [36], and others. Pal et. al used the FS-CCSD for applications to VIP and low-lying EEs for a number of small molecules [30,31,32,33]. Kaldor and co-workers similarly used FS-CC for atomic problems [34,35]. Related *equation-of-motion-CC* (EOM-CC) [39,40,41,42,43,44,45,46] was developed for IP, EA, and EE and it is especially worth noting that EOM-CC provides identical results of IP and EA as those obtained by the FS-CC for the (0.1) and (1.0) sector, respectively. However, FS-CC has an advantage that it is fully size-extensive, while in EOM-CC, another similarity transformation is required to bring the same effects [47,48].

In the quest to improve the results, it was important to introduce approximations to FS-CC, which have higher terms than singles and doubles. On an analysis by Mukherjee and Pal and co-workers [38,39], it was seen that the effective Hamiltonian, in singles and double approximations, misses terms originating from the triples and contributing perturbatively at the third order. Inspired by the above analysis, the triply excited cluster amplitudes were included in the FS (0.1) sector at the third order and later by Pal and co-workers at the fourth order [38]. Importance of triples in the context of EOM-CC was also noted in several studies for IP, EA, and EE [43,48].

The objective of this paper is to relook the fourth order perturbative triples inclusion. We present in this paper new partial fourth order schemes to FS-CCSD, which is also called MRCCSD, which may be useful for IP calculation. The main purpose is to show that one can design computationally less expensive partial fourth order triples schemes, which provide results in close agreement with the full fourth order method. Thus, essentially, a computationally efficient scheme in the context of fourth order triples to FS-CCSD will be presented.

The paper is presented as follows. In Section 2, we will introduce briefly the FS-CC, in particular, the (0.1) sector FS, which is the main context of this paper. Subsequently, we present the different fourth order schemes, from partial to the full, originating due to the triples; the corresponding bondonic-diagrammatic general formalism that phenomenological support the present partial fourth order triples schemes and further inspires the forthcoming perturbative many-body higher order chemical bonding dynamics is then innovatively advanced. Results and discussion section (Section 3) will highlight the main point of this paper, computationally the least expensive partial fourth order scheme, called the MRCCSD+$T^{*}-a\left(4\right)$ scheme, provides results of IP in agreement with full fourth order calculations in most cases. As one expects, the results of IPs are not very sensitive and the experimental numbers can only be reproduced with vibrational corrections. In our calculations presented here, the vibrational corrections are not included. In this section, third order triples results, full ($T^{*}\left(4\right)$) and another partial fourth order scheme $\left(T^{*}-b\left(4\right)\right)$ will also presented, such that a more detailed analysis can be made. We present results of outer-valence and inner-valence IPs of some test systems, N_2_, CO, BeO, and CH^+^.

## 2. Conceptual Method

### 2.1. FS-CC Theory

The common vacuum defines the set of holes and particles, which can be further sub-divided into “active” and “inactive” subsets. The reference in FS theory consists of determinants containing active particles and holes. In case determinants with all possible active holes and particles included in the model space, it is called a complete model space. If the reference consists of one active hole or one active particle only, the choice of active subsets can be always such that it is complete. A general *m-active* particle and *n-active* hole model space may be termed (*m*,*n*) model space. Such a reference $\Psi_{\mu }^{\left(0\right)\left[m,n\right]}$ may be written as:

(1) $$\Psi_{\mu }^{\left(0\right)\left[m,n\right]}=\overset{k}{\underset{i}{\sum }}c_{i\mu }\varphi_{i}^{\left[m,n\right]}\;,$$

where $|\varphi_{i}^{\left[m,n\right]}$ are *k*-number of determinants with *m* active particles and *n* active holes in the reference. The wave-operator, *Ω*, transforming $\Psi_{\mu }^{\left(0\right)}$ to $\Psi_{\mu }$ may be written as:

(2) $$\Omega =\left\{e^{{\tilde{s}}^{\left[m,n\right]}}\right\}\;,$$

where:

(3) $${\tilde{S}}^{\left[m,n\right]}=\overset{m}{\underset{k=0}{\sum }}\overset{n}{\underset{l=0}{\sum }}s^{\left(k,l\right)}.$$

Here, { } denotes the normal-ordering of the operators within the curly bracket. $s^{\left(k,l\right)}$ represents an operator destroying exactly *k* active particles and *l* active holes. Thus, ${\tilde{S}}^{\left[m,n\right]}$ consists of operators which can destroy up to *m* active particles and *n* active holes. The $s^{\left(k,l\right)}$ operators do not contract among themselves, since the *Ω* is normal ordered. In addition to the destruction of *k-active* particles and *l-active* holes, the cluster operators also create holes and particles involving inactive orbitals. This defines the total rank of the *S* operators. In singles and doubles approximations, one and two body cluster operators are used. Thus, the Bloch equations are partially decoupled and are solved from the (0.0) sector progressively upwards. The cluster operators are obtained through the Bloch equations at every FS sector, starting from (0.0) to (*m*,*n*). It can be shown that since the wave-operator is normal ordered, the equations for the lower sector are decoupled from the equations of the higher sector. This is called *sub-system embedding condition* (SEC) [49]. For the specific (0.1) sector, which is a complete model space, *intermediate normalization* (IN) is valid and the Bloch equation remains connected with IN [30,31]. For general model space, however, the connectivity of the Bloch equation is not consistence with the IN [28] and in such case, the IN condition is sacrificed. The IN is given as PΩ≡P. The Bloch equations for the specific (0,1) sector are:

(4) $$P^{\left(0,0\right)}\left(H_{N}\Omega -\Omega H_{eff}\right)P^{\left(0,0\right)}=0,$$

(5) $$Q^{\left(0,0\right)}\left(H_{N}\Omega -\Omega H_{eff}\right)P^{\left(0,0\right)}=0,$$

and:

(6) $$P^{\left(0,1\right)}\left(H_{N}\Omega -\Omega H_{eff}\right)P^{\left(0,1\right)}=0,$$

(7) $$Q^{\left(0,1\right)}\left(H_{N}\Omega -\Omega H_{eff}\right)P^{\left(0,1\right)}=0.$$

The first set of equations for (0.0) sector are, just the SRCC (*single reference coupled cluster*) equations. $H_{N}$ is the normal ordered Hamiltonian. Using the IN condition, the (0,1) effective Hamiltonian $H_{eff}^{\left(0,1\right)}$ can be defined as:

(8) $$P^{\left(0,1\right)}H_{eff}^{\left(0,1\right)}P^{\left(0,1\right)}=P^{\left(0,1\right)}{\left[H_{N}\Omega \right]}_{c}P^{\left(0,1\right)}\;.$$

The Schrodinger equation can be written for all roots corresponding to the number of determinants k in the reference space, as the eigen-value equation for $H_{eff}^{\left(0,1\right)}$ in $P^{\left(0,1\right)}$ space:

(9) $$\overset{k}{\underset{j=1}{\sum }}{\left\{H_{eff}^{\left(0,1\right)}\right\}}_{ij}c_{j\mu }=E_{\mu }c_{i\mu }{⍱}_{i}.$$

Due to the used of normal ordered Hamiltonian, $E_{\mu }$ is the correlation contributed to the $\mu^{th}$ state, computing with respect to the RHF of the N-electron. Further, if we compute ${\bar{H}}_{N}\equiv {\left[H_{N}e^{S^{\left(0,0\right)}}\right]}_{c}$ and drop ${\left(\bar{H}\right)}_{closed}$ in the construction of $H_{eff}^{\left(0,1\right)}$, the common correlation energy of the ground state is cancelled and we can obtain the direct difference energies. The earlier applications of FS-CC were done using singles and doubles approximations i.e.:

(10) $$S^{\left(0,0\right)}\sim S_{1}^{\left(0,0\right)}+S_{2}^{\left(0,0\right)},$$

(11) $$S^{\left(0,1\right)}\sim S_{1}^{\left(0,1\right)}+S_{2}^{\left(0,1\right)}.$$

This approximation is known as FS-CCSD approximation. The (0.0) sector operators are standard hole-particle creation operators, while (0,1) operators have one active hole destruction operator. $S_{1}^{\left(0,1\right)}$ operator must scatter from an inactive hole to an active hole and will thus be absent for a case where all holes are active:

(12) $$S_{1}^{\left(0,1\right)}=\underset{\beta \notin a_{h}}{\sum }\underset{\alpha \in a_{h}}{\sum }s_{\beta }^{\alpha }X_{\beta }^{†}X_{\alpha },$$

(13) $$S_{2}^{\left(0,1\right)}=\underset{\delta }{\sum }\underset{\gamma }{\sum }\underset{\beta }{\sum }\underset{\alpha \in a_{h}}{\sum }s_{\beta \delta }^{\alpha \gamma }\left\{X_{\beta }^{†}X_{\alpha }X_{\delta }^{†}X_{\gamma }\right\},$$

where $a_{h}$ refers to the subset of active holes.

### 2.2. Perturbative Triples

The full inclusion of triples is very expensive and may be unnecessary. Therefore, approximations have been proposed, motivated by perturbation. Perturbative triples were first proposed by Pal et al. [29]. In order to consider a balanced correlation for an entire wave function, the inclusion of both the $\;\hat{T}$_3_^(0,0)^ and $\;\hat{T}$_3_^(0,1)^ were considered. The corrections of the effective Hamiltonian at both third and fourth order due to triples were taken into account. To analyze the effect of perturbative triples to ${\;\hat{H}}_{eff}^{\left(0,1\right)},$ let us first consider the expression of ${\;\hat{H}}_{eff}^{\left(0,1\right)},$ including triples. First, let us write ${\bar{H}}_{N}$ as the sum of one-body, two-body, and three-body operators as:

$${\bar{H}}_{N}={\;\bar{f}}_{N}+{\;\bar{v}}_{N}+{\;\bar{w}}_{N}+\ldots .$$

Higher-body ${\bar{H}}_{N}$ will not be relevant for the discussion in this article. By definition, there are all open parts of $\bar{H}$, since the close part of $\bar{H}$ is dropped. This allows us to determine the difference of energy directly. Analysis of ${\;\hat{H}}_{eff}^{\left(0,1\right)}$ includes triples:

(14) $${\;\hat{H}}_{eff}^{\left(0,1\right)}={\;\hat{P}}^{\left(0,1\right)}\left\{{\;\bar{f}}_{N}+{\;\bar{f}}_{N}{\;\hat{S}}_{1}^{\left(0,1\right)}+{\;\bar{f}}_{N}{\;\hat{S}}_{2}^{\left(0,1\right)}+{\;\bar{v}}_{N}{\;\hat{T}\;S}_{2}^{\left(0,1\right)}+{\;\bar{v}}_{N}{\;\hat{S}}_{3}^{\left(0,1\right)}\right\}{\;\hat{P}}^{\left(0,1\right)}\;.$$

The equation for ${\;\hat{S}}_{3}^{\left(0,1\right)}$ can be written up to second order as:

(15) $${\;\hat{Q}}_{3}^{\left(0,1\right)}\left\{{\;\bar{f}}_{N}{\;\hat{S}}_{3}^{\left(0,1\right)}+{\;\bar{v}}_{N}{\;\hat{S}}_{2}^{\left(0,1\right)}+{\;\bar{w}}_{N}-{\;\hat{S}}_{3}^{\left(0,1\right)}{\;\bar{f}}_{N}\right\}{\;\hat{P}}^{\left(0,1\right)}=0.$$

We recall that correlation required for perturbation corrections, ${\bar{H}}_{N}$ will now contain ${\;\hat{S}}_{3}^{\left(0,0\right)}$, at the right order. In fact,$\;{\;\hat{S}}_{3}^{\left(0,0\right)}$, computed with $v_{N}{\;\hat{S}}_{2}^{\left(0,0\right)}$ with the energy denominator from the SRCC equation, suffices;${\;\bar{w}}_{N}$ constructed up to the second order, using ${\left(v_{N}{\;\hat{S}}_{2}^{\left(0,0\right)}\right)}_{L}$ is what is ${\bar{w}}_{N}^{\left[2\right]}$. For the rest of the terms, we use the converged values of ${\;\hat{S}}_{2}^{\left(0,1\right)}$ amplitudes. The corrected ${\;\hat{S}}_{2}^{\left(0,1\right)}$ amplitudes, at the second order, when inserted in Equation (14), provide what is called MRCCSD+$T^{*}$(3). This also implies that while ${\;\hat{S}}_{3}^{\left(0,1\right)}$ is corrected up-to the second order, partial higher order corrections are also taken care due to higher order effects on ${\;\hat{S}}_{2}^{\left(0,1\right)}$ from the MRCCSD equation.

To explain the various fourth order schemes, first, we include the effects of ${\hat{S}}_{3}^{\left(0,1\right)}$, calculated up-to 2nd order from Equation (15) in the ${\;\hat{S}}_{2}^{\left(0,1\right)}$ amplitude equation. This results in additional changes of ${\hat{S}}_{2}^{\left(0,1\right)}$ at third order:

(16) $${\;\hat{Q}}_{2}^{\left(0,1\right)}\left\{{\;\bar{f}}_{N}{\;\hat{S}}_{2}^{\left(0,1\right)}+{\;\bar{v}}_{N}+{\;\bar{v}}_{N}{\;\hat{S}}_{2}^{\left(0,1\right)}+{\;\bar{v}}_{N}{\;\hat{S}}_{3}^{\left(0,1\right)}+{\;\bar{w}}_{N}{\;\hat{S}}_{2}^{\left(0,1\right)}-{\;\hat{S}}_{2}^{\left(0,1\right)}{\;\hat{H}}_{eff}^{\left(0,1\right)}\right\}{\;\hat{P}}^{\left(0,1\right)}=0.$$

Clearly, this will affect the effective Hamiltonian at the fourth order and this scheme has been called MRCCSD+$T^{*}-a\left(4\right)$, which is a partial fourth order correction due to the triples. Hence, this scheme only includes changes in ${\;\hat{S}}_{2}^{\left(0,1\right)}$ equation at the third order due to ${\;\hat{S}}_{3}^{\left(0,1\right)}$ amplitudes. Clearly, this comes only from the second order ${\;\hat{S}}_{3}^{\left(0,1\right)}$ amplitudes, as are used in the T*(3) scheme.

Subsequently, we use ${\bar{w}}_{N}$ to at least the third order with the term ${v\;\hat{S}}_{3}^{\left(0,0\right)}$ as well as the new values of ${\;\hat{S}}_{2}^{\left(0,1\right)}$, as obtained in the MRCCSD+$T^{*}-a$(4) scheme, to calculate ${\;\hat{S}}_{3}^{\left(0,1\right)}\;\mathrm{amplitudes}\;\mathrm{to}\;\mathrm{the}\;\mathrm{third}\;\mathrm{order}$. The resulting equation is as follows:

(17) $${\;\hat{Q}}_{3}^{\left(0,1\right)}\left\{{\;\bar{f}}_{N}{\;\hat{S}}_{3}^{\left(0,1\right)}+{\;\bar{v}}_{N}{\;\hat{S}}_{2}^{\left(0,1\right)}+{\;\bar{w}}_{N}^{\left[3\right]}-{\;\hat{S}}_{3}^{\left(0,1\right)}{\;\bar{H}}^{\left(0,1\right)}\right\}{\;\hat{P}}^{\left(0,1\right)}=0.$$

In contrast to Equation (15), ${\;\bar{w}}_{N}$ is used up to the third order. This partially corrects ${\;\hat{S}}_{3}^{\left(0,1\right)}$ up-to the third order. It is important to emphasize that up-to this stage, the corrections in terms of triples are essentially non-iterative. Effective Hamiltonian, generated at this level, is still only partially correct up to the fourth order. This, we call MRCCSD+$T^{*}-b\left(4\right)$. To highlight the difference between *a*(4) and *b*(4) schemes due to the triples, *a*(4) includes changes in ${\;\hat{S}}_{2}^{\left(0,1\right)}$ amplitudes at the third order, keeping ${\;\hat{S}}_{3}^{\left(0,1\right)}\;\mathrm{amplitudes}\;\mathrm{at}\;\mathrm{the}\;\sec\mathrm{ond}\;\mathrm{order},$ while in *b*(4) scheme, additional corrections are made ${\mathrm{to}\;\mathrm{have}\;\hat{S}}_{3}^{\left(0,1\right)}$ partially corrected via Equation (17).

Finally, the term ${\bar{v}}_{N}{\;\hat{S}}_{3}^{\left(0,1\right)}$ with the second order ${\;\hat{S}}_{3}^{\left(0,1\right)}$ has been included (one iteration) in $Q_{3}^{\left(0,1\right)}$ equation to obtain the ${\;\hat{S}}_{3}^{\left(0,1\right)}$ values, which are correct up to third order (${\;\hat{S}}_{3}^{\left(0,1\right)}{}^{\left[3\right]})$:

(18) $${\;\hat{Q}}_{3}^{\left(0,1\right)}\left\{{\;\bar{f}}_{N}{\;\hat{S}}_{3}^{\left(0,1\right)}+{\;\bar{v}}_{N}{\;\hat{S}}_{2}^{\left(0,1\right)}+{\;\bar{w}}_{N}^{\left[3\right]}+v_{N}{\;\hat{S}}_{3}^{\left(0,1\right)}-{\;\hat{S}}_{3}^{\left(0,1\right)}{\;\bar{H}}^{\left(0,1\right)}\right\}{\;\hat{P}}^{\left(0,1\right)}=0.$$

The consequent ${\;\hat{H}}_{eff}$ is correct at least up to the fourth order. This final approximation is known as MRCCSD+$T^{*}\left(4\right)$. Effects of ${\;\hat{S}}_{3}^{\left(0,0\right)}$ in the construction of ${\bar{H}}_{N}$ have been taken to obtain the desired order correction.

### 2.3. Comparison with the Bondonic Diagrammatic Formalism of Many-Body Perturbation Theory

**Figure 1:**
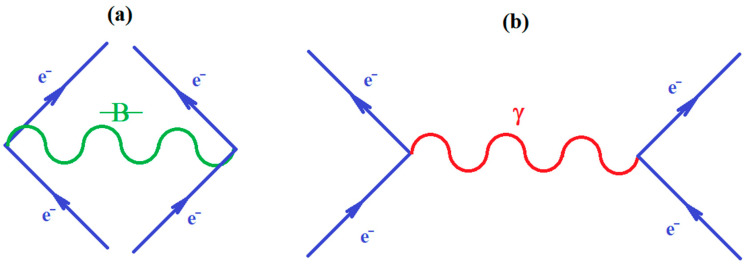
(**a**) The Feynman like diagram of inter-electronic pairing through the “gluing bondon” in chemical bonding; (**b**) The standard Feynman diagram of inter-electronic repulsion through “exchanging the photon” in free state.

**Figure 2:**
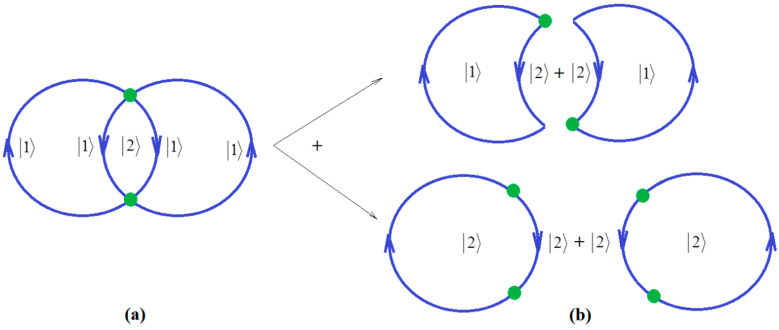
(**a**) The Bondonic graph of chemical bonding by electronic pairing formation; (**b**) The decomposition of bondonic graph as a superposition of active-holes and active hole-particles pairs, respectively.

Since ionization potential issue is closely related with molecular stability, through correlation, so with chemical bonding too, the natural additional matter may address how the quantum chemical bonding ultimate theory may accommodate the present perturbative many-body high-order schemes, in a general framework that may be eventually further developed. Fortunately, the bondonic theory of chemical bonding [6,7] may address this matter in a phenomenological way. To unfold this venture, one may start with the quantum electro-dynamically (Feynman) diagrams of chemical bonding, having the bondons as “gluing bosons” of electrons in bonding (Figure 1a), in analogy with the photons driving the free inter-electronic repulsion (Figure 1b). Starting from it, one may proceed with the next phenomenological step towards proposing the chemical bonding diagram of Figure 2a, while recognizing it is a superimposed of two interacting loops—in the decomposed version of Figure 2b.

Note that in Figure 2, due to the bosonic character of bondons in chemical bonding modeling, the interaction lines are mixed with the interaction centers, that is the single particle can self-interact in the first order and interact at distance in the second order, respectively for the mixed states |1〉 and |2〉. Accordingly, we may advance the bondonic graph (de)composition for electronic pairing in chemical bonding as the following:

(19) B[2](1)=2−(1+2)[2(12)+2(22)]

where we identified (from Figure 2b) the individual perturbative first and second order graphs, namely the active-hole and the active hole-particle pair, respectively. The prefactors of Equation (19) we better interpret when we provide its generalized form, i.e., for the *N*-body *k*-order of interaction:

(20) B[N](k)=2−(k+l)[N(kN)+N(k+1N)] =N22−(k+l)[(k1)+(k+11)]

This way: *k*- stay for the perturbation order; *l*- accounts for the total number of loops over all diagrams involved $\left(\begin{array}{c} k\\ 1 \end{array}\right),\left(\begin{array}{c} k+1\\ 1 \end{array}\right)$ - see Ref. [50] for special realization of such diagrams up to the fourth order, for instance; *N*- is the total number of electrons in the bonding state in matter (it can be either ground state or valence state, or other involved in chemical reactivity therefore). Of course Equation (20) is not “a derivation”, yet it has a phenomenological consistency, since: *i)* it carries the “effective formalism” feature by involving the summation (superimposing) of diagrams “each centered” on mate-/pairing- contributing active holes, as it is 
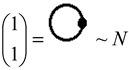
, along the higher interaction with active particles (k>1), as the effective formalisms usually prescribe; *ii)* it features the “superposition” multiplied with the $\sim N^{2}$ recalling the undiscernible particle statistics; *iii)* it is a particle size dependent as $\sim N^{2}$ so “recuperating” in a bosonic way (viz. the bondons as “gluing” the fermions in chemical bonding) the “condensation ordering parameter”, and being in the same time suitable for chemical bonding dynamics – since chemical reactivity usually conveys with $\sim N^{2}$ energy variation (e.g. by the chemical hardness dependency, etc.), see Ref. [51]. Moreover, worth mentioning that earlier study of applying bondonic theory to elemental chemical bonding in hydrogen molecule revealed that, indeed, the presence of $\sim N^{2}$ order parameter in the master quantum equation (of Hartree–Fock–Bogoliubov type) development; while, when combining with fermionic superposition in a Heitler–London formalism leaves with the shifts of both nominator and denominators of resulted variational energies of bonding, see Ref. [52]; this is in phenomenological agreement with the many-body perturbation theory which, through infinite summation of interacting orders yields with geometrical series solved with such energetic corrections in both effective potential and in referential energies alike. Of course, much work should be done in order to establish one-to-one correspondence between the present bondonic formalism as diagrammatic results and the standard perturbative theory of *N*-states. Yet, the present endeavors like to open such a way. All-in-all, as a corollary for the theoretical purpose of the actual paper, the bondonic diagrammatic framework of the present partial fourth order triples scheme looks like the composed diagram:

(21) B[1](α)×B[2](β)×B[3](γ)×(11)~N7

Expression (21) fulfils the current approximation scaling approach, while allowing specific realization (viz. the orders α,β,γ) as depending on the implemented scheme; particularly, the presently implemented schemes are represented by the bondonic composed diagrams, respectively:

(22) Eq. (14)/(15): MRCCSD+T*(3) …B[1](3)×B[2](2)×B[3](2)×(11)

(23) Eq. (16): MRCCSD+T*(3)−a(4) … B[1](3)×B[2](3)×B[3](2)×(11)

(24) Eq. (17): MRCCSD+T*(3)−b(4) …B[1](3)×B[2](2)×B[3](3)×(11)

(25) Eq. (18): MRCCSD+T*(4) … B[1](3)×B[2](3)×B[3](3)×(11)

One remarks the elegancy in expressing each of the above schemes in coding of the one-body, two-body, and three-body “bondonic operators”, in various orders of interactions, and with the reference to the active-hole creation. 

### 2.4. Computational Details

In this section, we present prototype results of VIP using the formulation of partial fourth order schemes for inclusion of triples in FS-CCSD. We chose four molecules, $N_{2}$, CO, BeO, and CH^+^ in different basis-sets. For simplicity, in this and the subsequent section, we simply write IP in place of VIP. We present MRCCSD, MRCCSD+$T^{*}$ (3), MRCCSD+$T^{*}-a\left(4\right)$, MRCCSD+$T^{*}-b\left(4\right),$ as well as full MRCCSD+$T^{*}\left(4\right)$ results for each of the molecules. We present the details of geometry, active orbitals, and the basis sets used for each molecule below.

For $N_{2}$, the two basis sets used are a [5s4p2d1f] (basis-A) and aug-cc-pVDZ basis (basis-B). Experimental geometry of 2.07 a.u. has been used for the calculations. $N_{2}$ is a well-tested molecule for which earlier FS-CCSD results are available. Basis-A has been generated by contracting 11 primitive s-type and 6 primitive p-type Gaussians to 5s and 40. Uncontracted 2d and 1f functions are added. The entire basis has been included in the Supplementary Information. Two active holes, $3\sigma_{g}$ and $1\pi_{u\;}\left(\mathrm{in}\;\mathrm{actual}\;\mathrm{calculation},\;\mathrm{three},\;\sin\mathrm{ce}\;\pi \;\mathrm{is}\;a\;\mathrm{doubly}\;\mathrm{degenerate}\;\mathrm{orbital}\right),$ are used as active.

For CO, the results have been computed with three different basis sets, cc-pVDZ, cc-pVTZ, and augmented cc-pvDZ basis sets. Experimental bond distance of 2.132 a.u. has been used for the calculations. We chose four active holes, 5σ, 1*π* (double degenerate), 4σ for the calculations.

For BeO, we took *π* and σ orbitals, which are the two highest occupied molecular orbitals. cc-pVDZ, cc-pVTZ, and augmented cc-pvDZ basis sets were used. Experimental geometry of 2.515 a.u has been used to present vertical IPs using the above methods.

Finally, a small molecule, CH^+^, has been taken for study where two highest molecular orbitals have been taken as active. In this case, however, we presented the lowest IP only. The calculation has been done using cc-pVDZ and cc-pVTZ basis at a bond distance of 1.8 a.u., 1.9 a.u., and 2.0 a.u. The IP calculations for CH^+^ was earlier used for discussing binding of CH ^2+^ [53,54].

The molecules and basis sets have been chosen such that the conclusion can be drawn on a verity of things. A few cases have been chosen with augmentation of basis to explore the effects of diffuse functions on vertical IPs.

## 3. Results and Discussions

First, we look at the results of IPs of $N_{2}$ in the two bases (Table 1). We compare the results using perturbative triples at third and fourth order. We find that in the basis-A, the results for both $3\sigma_{g}$ and $1\pi_{u}$ decrease with triples at third order and then oscillate. In this basis, we see not so significant effect of fourth order correlation due to the triples. However, what is more important is that $T^{*}-a\left(4\right)$, which is computationally the least expensive method and $T^{*}-b\left(4\right)$ can be regarded to be satisfactory. Due to the oscillating nature, it is difficult to judge the quality of the approximations. This is true for both $3\sigma_{g}$ and $1\pi_{u}$ IPs. When we examine the results of IP in other basis, called basis-B, we find that $3\sigma_{g}$ and $1\pi_{u}$ IPS have differing trends. $3\sigma_{g}$ IP increases with the triples and then typically at different partial fourth order schemes oscillates. On the other hand, $1\pi_{u}$ IP decreases with the triples at third order before having oscillatory trends at partial fourth order schemes. One of the differences between the two bases is the presence of diffuse functions in basis-B. It is likely that the diffuse functions affect the $3\sigma_{g}$ and $1\pi_{u}$ differently as we add the triples and can be the cause for varying trends. The results in both the basis agree well with the experiments.

As a next example, we consider CO molecule. Three different basis sets have been used. The results are presented in Table 2. We find in all the three bases, the lowest IP i.e., one which is ionized from the 5σ orbital, decreases slightly at $T^{*}$ (3) level from MRCCSD results and then oscillates at different partial fourth order levels. On the other hand, the other two inner valence IPs 1*π* and 4σ, have different trends at the third order level. It increases and then as in the earlier cases of triples at the fourth order levels, oscillates. A comparison with basis-A indicates that diffuse functions do not play a significant role in this case and neither does it change the trends of the three IPs.

For BeO, the highest MO is of *π* symmetry. The IPs of the highest 1*π* and the nest highest 4σ orbital are given in the Table 3.

We see in this case the larger effect of triples at third order. The IPs increase compared to the SD results and then the partial fourth order triples bring them back towards the FS-CCSD calculations. This is seen for all three cases. Comparing with the full results, we clearly see in this case, MRCCSD+$T^{*}-a\left(4\right)$ results are quite sufficient in providing results close to the full. $T^{*}-b\left(4\right)$ makes marginal changes. The trend is seen for all three basis sets, cc-pVDZ, cc-pVTZ, and aug-cc-pVDZ basis. Comparison of cc-pVDZ and aug-cc-pVDZ, we also find the negligible contribution of diffuse functions.

As a final example, we discuss IPs of CH^+^. There were earlier studies on the stability of CH-dication, many of which showed the repulsive nature of the bonding. Calculations by Wetmore et al. [53] using a multi-reference configuration interaction model with dzp basis showed a shallow potential well of a very small depth (0.01 eV) trapped behind a slight potential barrier in the ground state curve of CH^2^+. The dip was caused by a strong interaction with the second excited state (C^2+^ + H) of the same symmetry. It was, however, too shallow to explain and support the existence of a metastable dication and, in fact, disappeared with a slightly larger basis set. We recomputed with somewhat larger basis using the FS-CC method and observed a very small dip [5Total energy of CH-dication was computed by adding the IP value to the SRCC ground sate energy of CH^+^. In that sense, the calculation of IP was used to explain the binding of a radical. The calculation of potential energy surface (PES), however, needs theories, which are of Hilbert-space type, but FS theory can be used to throw some light. In this example, we do not wish to generate PES, but calculate the lowest IP of CH^+^ at three bond distances to see how they change with the triples using cc-pVTZ and cc-pVDZ basis. The results are presented in Table 4 and Table 5, respectively. We see from Table 4 that the IP for each of the distances decreases with the triples at the third order as well as various partial fourth order schemes, compared with the MRCCSD. The oscillating character of the partial fourth order schemes is not seen in this case. This indicates that energy of the CH-dication will be less as triples are added to the MRCCSD. This, however, does not indicate stronger binding, since we have not computed the full PES, computation of which is outside the scope of the present paper. Further, the results of IP decrease with stretching from 1.8 a.u. to 2.0 a.u. For the cc-pVDZ basis reported in Table 5, we find similar trends, except that there is a slight oscillation between MRCCSD +T* − *a*(4) and T* − *b*(4) results. For this small basis, for comparison, we have presented full CI results. The general agreement is observed. What is of significance, however, is that $T^{*}$ − *a*(4), which is computationally far less expensive, is sufficient for this case.

We now look at the computational cost of different fourth order schemes. We note that the schemes of third order and fourth order triples are calculated sequentially in the order MRCCSD+T*(3), MRCCSD+T* − *a*(4), MRCCSD+T* − *b*(4), and MRCCSD+T*(4). Naturally computing times progressively go up. All of these scale as N^7^. However, third order triples are calculated first and thus run fast. These also have very few diagrams. Essentially, it means that the prefactor is quite small. The next scheme computed is $T^{*}$ − a (4), followed by $T^{*}$ − *b*(4) and full fourth order. Clearly, the computational time required goes in the same order. However, it is important to note that in terms of diagrams, the two latter schemes $T^{*}$ − *b*(4) and full fourth order have more in number. Typical computing time to calculate T*(4) takes at least 2 to 3 times the time that is taken for T* − *a*(4) for the molecules that we presented. This will scale even worse as we go for larger molecules. Exact computing times are not relevant yet, since the code is unoptimized. The prefactor for coding the diagrams of T* − *a*(4) is much smaller compared to the prefactor for additional diagrams of T* − *b*(4). Although T* − *a*(4) results, by themselves, are not sufficient, these will still turn out to be computationally optimum. Hence, we conclude that this itself is a promising candidate for approximate inclusion of fourth order triples from the efficacy of computational time. However, the approximations MRCCSD+T* − *a*(4) and MRCCSD+T* − *b*(4) have the limitations in reproducing the full fourth order values, as is seen in the cases of N_2_ and CO.

## 4. Conclusions and Perspectives

Analysis of the results has pointed out that the MRCCSD+$T^{*}-a\left(4\right)$ scheme, has come out as a promising candidate a search of computationally least expensive partial fourth order scheme. With some limitations, it provides results in agreement with the full fourth order and experiments. Moreover, the present partial fourth order triples schemes stimulate the advancing of the diagrammatic bondonic formalism featuring the compact representation of the chemical bond dynamics, here applied on vertical ionization schemes, while opening further challenging in treating exotic or bigger molecules with the aid of diagrammatic perturbation theory for many-states.

## Figures and Tables

**Table 1:** Vertical ionization potentials of N_2_ using various basis sets.

METHODS	Orbital	RESULTS (eV)	
		Basis-A (5s4p2d1f)	Basis-B (aug-cc-pVDZ)
MRCCSD	3$\sigma_{g}$	15.645	15.443
	$1\pi_{u}$	17.262	17.129
MRCCSD+$T^{*}\left(3\right)$	3$\sigma_{g}$	15.637	15.486
	$1\pi_{u}$	16.966	16.890
MRCCSD+$T^{*}-a\left(4\right)$	3$\sigma_{g}$	15.280	15.189
	$1\pi_{u}$	16.750	16.685
MRCCSD+$T^{*}-b\left(4\right)$	3$\sigma_{g}$	15.639	15.525
	$1\pi_{u}$	17.000	16.908
MRCCSD+$T^{*}\left(4\right)$	3$\sigma_{g}$	15.541	15.424
	$1\pi_{u}$	16.907	16.810
Experimental Ref. [55]	3$\sigma_{g}$	15.581 ± 0.008	
	$1\pi_{u}$	16.8	

**Table 2:** Vertical ionization potentials of CO using various basis sets.

METHODS	Orbital	RESULTS (eV)		
		Basis-A (cc-pVDZ)	Basis-B (cc-pVTZ)	Basis-C (aug-cc-pVDZ)
MRCCSD	5$\sigma_{g}$	13.827	14.149	13.995
	$1\pi_{u}$	16.746	17.048	16.915
	4$\sigma_{u}$	19.487	19.759	19.678
MRCCSD+$T^{*}\left(3\right)$	5$\sigma_{g}$	13.616	13.967	13.837
	$1\pi_{u}$	16.847	17.137	17.092
	4$\sigma_{u}$	20.030	20.274	20.293
MRCCSD+$T^{*}-a\left(4\right)$	5$\sigma_{g}$	13.458	13.763	13.642
	$1\pi_{u}$	16.559	16.773	16.769
	4$\sigma_{u}$	19.170	19.357	19.498
MRCCSD+$T^{*}-b\left(4\right)$	5$\sigma_{g}$	13.663	13.988	13.865
	$1\pi_{u}$	16.766	17.026	16.995
	4$\sigma_{u}$	19.424	19.653	19.769
MRCCSD+$T^{*}\left(4\right)$	5$\sigma_{g}$	13.575	13.903	13.773
	$1\pi_{u}$	16.716	16.968	16.936
	4$\sigma_{u}$	19.336	19.554	19.671
Experimental Ref. [56]	5$\sigma_{g}$	14.0142 ± 0.0003		
	$1\pi_{u}$	16.5		
	4$\sigma_{u}$	19.7		

**Table 3:** Vertical IPs of BeO using various basis sets.

METHODS	Orbital	RESULTS (eV)		
		Basis-A (cc-pVDZ)	Basis-B (aug-cc-pVDZ)	Basis-C (cc-pVTZ)
MRCCSD	$1\pi_{u}$	9.786	9.927	9.921
	4$\sigma_{u}$	10.816	11.005	10.962
MRCCSD+$T^{*}\left(3\right)$	$1\pi_{u}$	10.358	10.529	10.429
	4$\sigma_{u}$	11.681	11.879	11.744
MRCCSD+$T^{*}-a\left(4\right)$	$1\pi_{u}$	9.646	9.648	9.649
	4$\sigma_{u}$	10.539	10.556	10.540
MRCCSD+$T^{*}-b\left(4\right)$	$1\pi_{u}$	9.888	9.909	9.934
	4$\sigma_{u}$	10.810	10.853	10.859
MRCCSD+$T^{*}\left(4\right)$	$1\pi_{u}$	9.760	9.800	9.934
	4$\sigma_{u}$	10.607	10.684	10.663
Experimental Ref. [57]	$1\pi_{u}$	10.10		
	4$\sigma_{u}$	10.40		

**Table 4:** Vertical IP of CH^+^ (lowest) in cc-pVTZ basis in eV.

IP (eV)	Methods				
Bond Distance (a.u.)	MRCCSD	$\mathrm{MRCCSD}+T^{*}\left(3\right)$	$\mathrm{MRCCSD}+T^{*}-a\left(4\right)$	$\mathrm{MRCCSD}+T^{*}-b\left(4\right)$	$\mathrm{MRCCSD}+T^{*}\left(4\right)$
1.8	28.580	28.363	28.040	28.042	27.865
1.9	28.267	28.057	27.657	27.650	27.470
2.0	27.396	27.734	27.256	27.240	27.058

**Table 5:** Vertical IP of CH^+^ (lowest) in cc-pVDZ basis in eV.

IP (eV)	Methods					
Bond Distance (a.u.)	MRCCSD	$\mathrm{MRCCSD}+T^{*}\left(3\right)$	$\mathrm{MRCCSD}+T^{*}-a\left(4\right)$	$\mathrm{MRCCSD}+T^{*}-b\left(4\right)$	$\mathrm{MRCCSD}+T^{*}\left(4\right)$	Full CI (FCI)
1.8	24.594	24.581	24.550	24.575	24.549	24.528
1.9	24.424	24.409	24.377	24.403	24.376	24.354
2.0	24.245	24.230	24.195	24.224	24.196	24.189

## Supplementary Materials

Supplementary materials can be found at https://www.mdpi.com/1422-0067/21/17/6199/s1.
