# Supplementary material for: Fock-Space Coupled Cluster Theory: Systematic Study of Partial Fourth Order Triples Schemes for Ionization Potential and Comparison with Bondonic Formalism
Source: Int J Mol Sci. 2020 Aug 27;21(17):6199. doi: 10.3390/ijms21176199 (PMC7504140; doi:10.3390/ijms21176199)
Supplement: Supplementary file 1 [file ijms-21-06199-s001.pdf]

International Journal of *Molecular Sciences*

MDPI

Supplementary Information

Fock-Space Coupled Cluster Theory: Systematic Study of Partial Fourth order Triples Schemes for Ionization Potential and Comparison with Bondonic Formalism

Suhita Basumallick [1], Sourav Pal [1,2,*] and Mihai V. Putz [3,4,*]

[1] Department of Chemistry, Indian Institute of Technology Bombay, Powai, Mumbai 400076, India; basumallick.suhita16@gmail.com
[2] Department of Chemical Sciences, Indian Institute of Science Education and Research Kolkata, Mohanpur, Nadia 741246, West Bengal, India
[3] Laboratory of Structural and Computational Physical-Chemistry for Nanosciences and QSAR, Biology-Chemistry Department, Faculty of Chemistry, Biology, Geography, West University of Timisoara, Pestalozzi Street No. 16A, 300115 Timisoara, Romania
[4] Laboratory of Renewable Energies-Photovoltaics, R&D National Institute for Electrochemistry and Condensed Matter, Street Dr. Aurel Podeanu No. 144, 300569 Timisoara, Romania
* Correspondence: s.pal@iiserkol.ac.in (S.P.); mihai.putz@e-uvt.ro (M.V.P.); Tel.: + 91-33-6136-0012 (S.P.); +40-0256-592-638 (M.V.P.)

Received: 06 August 2020; Accepted: 25 August 2020; Published: date

Nitrogen Molecule BASIS A (Gamess Format)

```
Nitrogen    7.0     0.000      0.000      1.035
S   8
  1  11420.0000000          0.0005230
  2   1712.0000000          0.0040450
  3    389.3000000          0.0207750
  4    110.0000000          0.0807270
  5     35.5700000          0.2330740
  6     12.5400000          0.4335010
  7      4.6440000          0.3474720
  8      0.5118000         -0.0085080
S   8
  1  11420.0000000         -0.0001150
  2   1712.0000000         -0.0008950
  3    389.3000000         -0.0046240
  4    110.0000000         -0.0185280
  5     35.5700000         -0.0573390
  6     12.5400000         -0.1320760
  7      4.6440000         -0.1725100
  8      0.5118000          0.5999440
S   1
  1      1.2930000          1.0000000
S   1
```

International Journal of
Molecular Sciences

1	0.1787000	1.0000000
S	1	
1	0.0576000	1.0000000
P	3	
1	26.6300000	0.0146700
2	5.9480000	0.0917640
3	1.7420000	0.2986830
P	1	
1	0.5550000	1.0000000
P	1	
1	0.1725000	1.0000000
P	1	
1	0.0491000	1.0000000
D	1	
1	1.6540000	1.0000000
D	1	
1	0.4690000	1.0000000
F	1	
1	1.0930000	1.0000000
